# Personalising cardiovascular network models in pregnancy: A two‐tiered parameter estimation approach

**DOI:** 10.1002/cnm.3267

**Published:** 2020-01-13

**Authors:** Jason Carson, Lynne Warrander, Edward Johnstone, Raoul van Loon

**Affiliations:** ^1^ Zienkiewicz Centre for Computational Engineering, College of Engineering Swansea University Swansea UK; ^2^ Data Science Building, Swansea University Medical School Swansea University Swansea UK; ^3^ HDR UK Wales and Northern Ireland Health Data Research UK London UK; ^4^ Maternal and Fetal Health Research Centre, Division of Developmental Biology, Faculty of Medicine Biology and Health University of Manchester Manchester UK

**Keywords:** parameter estimation, personalised haemodynamic model, pre‐eclampsia, pregnancy, uterine artery waveform

## Abstract

Uterine artery Doppler waveforms are often studied to determine whether a patient is at risk of developing pathologies such as pre‐eclampsia. Many uterine waveform indices have been developed, which attempt to relate characteristics of the waveform with the physiological adaptation of the maternal cardiovascular system, and are often suggested to be an indicator of increased placenta resistance and arterial stiffness.

Doppler waveforms of four patients, two of whom developed pre‐eclampsia, are compared with a comprehensive closed‐loop model of pregnancy. The closed‐loop model has been previously validated but has been extended to include an improved parameter estimation technique that utilises systolic and diastolic blood pressure, cardiac output, heart rate, and pulse wave velocity measurements to adapt model resistances, compliances, blood volume, and the mean vessel areas in the main systemic arteries. The shape of the model‐predicted uterine artery velocity waveforms showed good agreement with the characteristics observed in the patient Doppler waveforms. The personalised models obtained now allow a prediction of the uterine pressure waveforms in addition to the uterine velocity. This allows for a more detailed mechanistic analysis of the waveforms, eg, wave intensity analysis, to study existing clinical indices. The findings indicate that to accurately estimate arterial stiffness, both pulse pressure and pulse wave velocities are required. In addition, the results predict that patients who developed pre‐eclampsia later in pregnancy have larger vessel areas in the main systemic arteries compared with the two patients who had normal pregnancy outcomes.

## Graphical abstract

Cardiovascular network models are personalised by optimising toward a range of noninvasive measurements during pregnancy, such as patient height, heart rate, blood pressure, cardiac output, and pulse wave velocity. The developed framework was tested on data from four pregnant patients, and the resulting model‐predicted uterine wave forms share the same characteristics as those measured. This modelling approach allows a more mechanistic analysis of flow and pressure in pregnancy and might provide alternative powerful biomarkers for pre‐eclampsia.

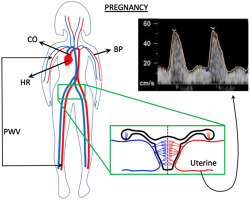

## 1 INTRODUCTION

During a healthy pregnancy, significant physiological adaptations occur to the structure and function of the cardiovascular system. There are increases in cardiac output (CO) and blood volume by approximately 40*%*,^1^, ^2^ a decrease in total peripheral resistance by up to 30*%*, and an increase in arterial compliance by approximately 35*%*.^3^ The mean arterial blood pressure normally decreases over the first and second trimesters before rising again in the third trimester, close to term.^4^ The blood supply to several organs, particularly the kidneys and uterus, substantially increases. The creation of the placenta occurs to facilitate nutrient transport exchange between the maternal and foetal systems, which also helps in reducing the vascular resistance of the uterine region, and thus significant increases in blood supply to the uterus are observed over the course of pregnancy. The left and right uterine arteries are the largest of the vessels that supply blood to the uterus. It has been observed that the uterine artery can more than double in diameter during a healthy pregnancy, which helps accommodate the increased demands of blood supply of the uterine region.^5^

Due to the number of physiological adaptations that the maternal system is required to undergo, there are many pathologies which can develop as a result of insufficient adaptation.^2^ There have been many attempts to link various noninvasive measurements to several of these pathologies, such as mean arterial pressure,^4^, ^6^, ^7^ pulse pressure,^6^, ^8^, ^9^, ^10^ pulse wave velocity (PWV),^11^, ^12^, ^13^, ^14^ and augmentation index;^13^, ^15^, ^16^ although the use of the augmentation index as a measure of arterial stiffness has been criticised,^17^, ^18^ there are many factors involved in the creation of the augmentation pressure that occurs from the various wave‐reflections. The uterine artery velocity waveform is considered an important indicator for predicting and detecting the development of pathologies that may occur as a result of poor physiological adaptation in pregnancy.^19^, ^20^, ^21^, ^22^, ^23^ Doppler studies have been utilised to investigate: uterine vascular resistance changes,^24^, ^25^ compliance changes,^26^ uterine artery volumetric flow rate changes,^27^, ^28^, ^29^ pre‐eclampsia,^30^, ^31^, ^32^, ^33^, ^34^, ^35^ and hypertension and hypertensive disorders.^36^, ^37^, ^38^, ^39^ As a result, many Doppler indices have been proposed^33^ which take into account the shape and magnitude of the uterine artery velocity waveform.

Typically, models of human pregnancy consider either the foetal system,^40^, ^41^ or the maternal‐foetal interface that includes the placenta,^42^, ^43^ umbilical cord,^44^, ^45^ and a model which focuses on the foetal circulation but also includes the placenta and uterine arteries of the maternal system.^46^ A lumped model was proposed by Corsini et al^47^; however, the model considered only one pathway for blood supply to the uterus and thus it is not capable of capturing the complex flow behaviour that occurs in the uterine region. Recently, more comprehensive models have been developed, such as: the model by Clark et al^48^ that considers the uterine circulation and placenta in a lumped parameter model with a fixed inlet boundary condition, allowing an investigation of how the uterine artery velocity waveform depends on the uterine vasculature; the model by Carson et al^49^, ^50^ that considered a complex closed‐loop model of the entire female cardiovascular system that incorporated noninvasive patient measurements into the model via a parameter estimation technique. The model included 1D representations of the major vessels in the systemic and pulmonary, arteries, and veins, and lumped models representing the heart, valves, and vascular beds. Furthermore, the model included the two main pathways of blood supply to the uterus, the uterine arteries that branch from the internal iliac arteries to supply the uterus from below, and the utero‐ovarian communicating arteries that supply the uterus from above.

The work presented here places the developed closed‐loop model in an optimisation framework that is designed to create personalised models in pregnancy. For four pregnant patients, a range of noninvasive measurements, such as systolic blood pressure (SBP) and diastolic blood pressure (DBP) (brachial artery), heart rate (HR), CO, and PWV, are integrated into a computational modelling approach. This allows parameter estimations for both the more global systemic maternal arterial system and the more local utero‐ovarian system, and a comparison of the model‐predicted uterine waveforms with the measurements.

## 2 MATERIALS AND METHODS

In this section, an overview of the model of the entire female cardiovascular system is given. The initial and iterative parameter estimation techniques that are utilised in the model are described. The patient measurement data utilised in this work is then introduced, and a critical overview of spectral Doppler ultrasound is presented.

### 2.1 Haemodynamic “template” model of pregnancy

The haemodynamic model of pregnancy utilised as the basis for personalisation in this work has been previously validated and is described in detail in Carson et al.^49^, ^50^ The model extended the cardiovascular model of Mynard and Smolich^51^ by including vessels and organs in the utero‐ovarian region. As the model has been described in Carson et al,^50^ only the main components of the model are presented here. The model includes the following:

- Five hundred thirteen 1D vessel segments, including systemic arteries, hepatic portal veins, systemic veins, pulmonary arteries, and pulmonary veins.
- Sixty‐one 0D vascular beds, which include organs such as the heart, liver, brain, kidneys, ovaries, uterus, and placenta and also capillary systems to body regions and tissues such as the arms, legs, chest, and pelvis.
- A change of the unstressed vessel diameter and compliances of uterine arteries based on the gestational week.

In this work, only data from the systemic arterial system was available so the model parameters in the systemic venous and pulmonary systems are chosen to give healthy mean pressures of 5 mmHg in the systemic venous system and mean pulmonary arterial and venous pressures of less 25 and 12 mmHg, respectively.

**FIGURE 1:**
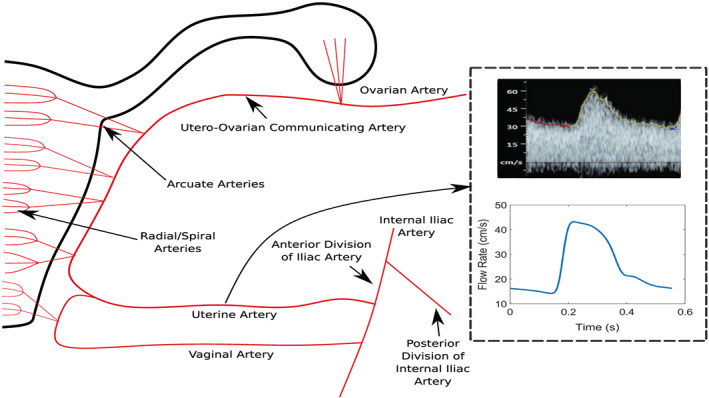
Location of uterine artery waveform shown in the results analysis from the model

The governing equations of the 1D and 0D models are solved using the implicit sub‐domain collocation scheme described in Carson and Van Loon.^49^, ^52^ For the 1D system, this involves a second‐order backward difference discretisation for the temporal terms and spatial integration is performed using the composite trapezoidal rule (for the 1D model). Figure 1 shows one side of the uterine arterial network implemented in this work.

### 2.2 Patient characteristics

Data was collected with NHS REC approval (11/NW/0426), and all patients were identified through the translational research clinics at St Mary's Hospital, Manchester, UK (Table 1). Diseased patients were retrospectively identified from a cohort of patients who developed early‐onset placental disease (both early‐onset foetal growth restriction and pre‐eclampsia) and required pre‐term delivery. Control pregnancies were identified from a patient cohort with chronic hypertension. Despite having chronic hypertension, no patient required antihypertensive treatment throughout pregnancy nor developed any pregnancy complications, and delivered spontaneously at term.

**TABLE 1:** Patient information and pregnancy outcome data

							Early	
	Maternal				Birth		Onset	
	Age	Past		GAD	Weight		Placental	Pregnancy
Patient	(years)	Medical History	Parity	(Week + Day)	(g)	Infant Gender	Disease	Outcome
1	37	Nil significant	3	29 + 3	495	Male	Yes	Neonatal death
2	28	Chronic hypertension	1	28 + 2	550	Male	Yes	Alive and well
3	37	Chronic hypertension	0	40 + 5	3670	Male	No	Alive and well
4	26	Chronic hypertension	0	40 + 2	3694	Female	No	Alive and well

*Note*. Parity represents the number of previous pregnancies that have reached gestation, GAD is the gestational age at delivery.

### 2.3 A remark on Doppler ultrasound

As uterine Doppler measurements will play a key role in both the personalisation processes of our cardiovascular models, but also in the reflective process when studying the model‐predicted waveform shapes, a few critical notes should be made to appreciate the limitations of such measurements. The shaded area in a Doppler (spectral) ultrasound represents the full spectrum of blood flow velocities in the vessel cross‐section at a specific axial location. Although the full frequency spectrum contains information on type of velocity profile, such as a narrow frequency shift corresponding to a flat/plug shaped profile, this information cannot readily be extracted with satisfactory levels of confidence.^53^

It is generally thought that the maximum frequency shift at a given time corresponds to the maximum velocity in the cross section. However, this depends strongly on the type of velocity profile within that cross‐section and due to intrinsic spectral broadening, this is not strictly true,^54^ which makes it difficult to rely on the magnitude of the velocities that are extracted via pulsed Doppler.

In addition, the volumetric flow rate extracted via Doppler is also subject to strong criticism^53^, ^55^, ^56^ when calculated from the maximum velocity. Velocity profiles change in time and space, and as such, the maximum velocity can be more than double the value of the cross‐sectional mean velocity.^53^, ^56^ Add to this the error in measurements of the vessel area and it is clear that volumetric flow estimations need to be treated with care.

Finally, the accuracy of spectral Doppler is also vulnerable to human factors,^57^, ^58^ of which one of the most important is the alignment of the transducer with respect to the axial direction of the vessel. A correction factor is applied to the velocity signal in order to try and account for this alignment, but even after application of this correction factor, Doppler tends to overestimate velocities. Angles greater than 60° are known to produce larger errors,^53^ and in the range 40 to 60° (generally the angle of operation), it has been observed that the estimated velocity changes by approximately 2.1*%* per degree, which is very significant.

In conclusion, it should be appreciated that there is significant uncertainty in the magnitude of the velocities measured by an ultrasound machine and, as a consequence, the reliability of most uterine artery waveform indices based on Doppler ultrasound should be treated with care. Hence, in this work, less emphasis will be given to the maximum velocity magnitudes while more attention will be given to the shape and characteristics of the velocity waveforms.

### 2.4 Parameter estimation

Parameter estimation is perhaps the most difficult part of modelling a complex model of the cardiovascular system as there may be many parameter combinations that could produce similar results. A parameter sensitivity test on the current model has been performed in Carson^49^ and showed that the model solutions are relatively insensitive to the cardiac parameters that are assumed.

The arterial network implemented in this work includes the uterine arteries that supply the uterus with blood originating from the internal iliac arteries and the utero‐ovarian communicating arteries that supply the uterus with blood from the descending aorta (and renal arteries) via the ovarian arteries. The utero‐ovarian network also includes the anastomosis between the uterine and utero‐ovarian communicating arteries. It is assumed that there are a total of 20 arcuate arteries that branch from the uterine arteries and a further 50 radial/spiral arteries that branch from the arcuate arteries. The geometry (length and diameters) of the vessels are primarily chosen based on values found in literature. As no information is known about the location of the placenta, the middle eight arcuate arteries and 200 spiral arteries (total of left and right side of the uterus) are chosen to supply blood to the placenta. Furthermore, it is assumed that the utero‐ovarian network is symmetric, so left‐ and right‐sided vessels have the same area and diameter.

**FIGURE 2:**
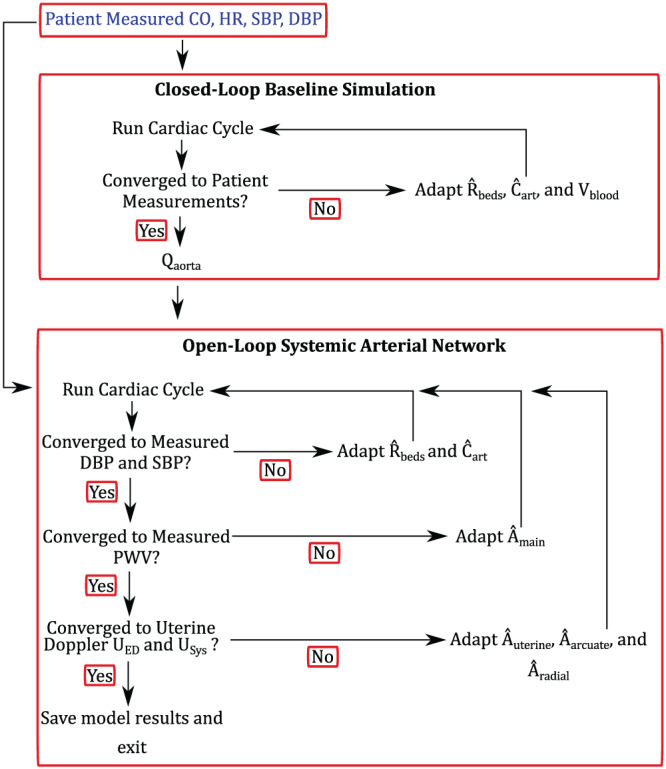
Overview of the two‐tiered parameter estimation algorithm

In this work, we utilise a two‐tiered parameter estimation algorithm, shown in Figure 2. The first tier involves a closed‐loop model that is described in previous studies.^49^, ^50^ The model utilises the patient‐specific,SBP, DBP, HR, and CO, for both the initial and iterative adaptation of model parameters that includes the adaptation of the peripheral resistances, ${\hat{R}}_{beds}$, the arterial compliances, ${Ĉ}_{art}$, and blood volume, *V*_*blood*_. After convergence in tier 1, the resulting flow waveform at the inlet of the aorta is fixed and prescribed as a Dirichlet boundary condition in tier 2. The resistances and compliances are kept as initial estimates for the second tier of the parameter estimation technique. Tier 2 involves adapting ${\hat{R}}_{beds}$ and ${Ĉ}_{art}$ together with the systemic arterial areas, ${Â}_{art}$, to achieve the same values for SBP, DBP, and HR, but also PWV. At the same time, the areas of the uterine arteries and arcuate arteries, ${Â}_{ut}$, together with the areas of the spiral arteries, ${Â}_{spiral}$, will be changed until the same peak systolic velocities, *U*_*sys*_, and end diastolic velocity values, *U*_*ED*_ are found as in the measured Doppler velocity waveforms. All simulations are performed until the models SBP and DBP (brachial artery) and CO are within 1*%* error of the measured data values and that the model solution at the end of a cardiac cycle is within 1*%* error of the model solution from the previous cardiac cycle. A more detailed description on the adaptation of the circulations is given next.

#### 2.4.1 Parameter estimation tier 1

The first iteration of the parameter estimation strategy has been described in detail previously.^49^, ^50^ The arterial system is of primary interest in this paper, and, hence, only the parameter estimation in the systemic arteries is described although the entire closed‐loop circulation model is considered in this first tier. The strategy involves utilising the following patient data to estimate the model parameters:

- The measured HR is used in the model, and the patient height is used to scale the lengths of all vessels.
- The initial total peripheral resistance is estimated using the patient's measured mean arterial pressure and CO.
- The distribution of this resistance to each vascular bed is determined by using a combination of the expected percentage of CO to each body region with a further splitting of these resistances into each vascular bed in that region, using Murray's law with an exponent of 2.76.
- The total arterial compliance distribution to each vascular bed is estimated using the characteristic time constant *τ*=*RC*=1.79. The compliance is then distributed to each vascular bed by first subtracting the 1D compliances from the total arterial compliance and then distributing inversely to that of the resistances.
- The blood volume in the system is iteratively added or subtracted over each cardiac cycle via the venules in order to change the CO in the model. This is performed until the CO of the model is within 1*%* of the patient measurements.

#### 2.4.2 Parameter estimation tier 2

The second tier of the parameter estimation strategy utilises the same parameters as the first tier with the following changes:

- The model is converted into an open‐loop systemic arterial network, ie, the venous system and heart model are not considered.
- The addition of blood volume to achieve the aimed CO is replaced by defining the volumetric flow rate in the aortic root from the first tier 1 as the inlet boundary condition, ie, the CO and flow waveform will be fixed.

Furthermore, two additional patient measurements are included for parameter estimation in this second tier, ie, (a) the PWV (in this case, the brachial‐ankle PWV) and (b) the uterine artery Doppler ultrasound waveform.

There are several equivalent analytical formula of PWV, such as the Moens‐Korteweg, and Bramwell‐Hill equations, respectively given by

(1) $$PWV=\sqrt{\frac{Eh}{2\rho r}},=\sqrt{\frac{A}{\rho C_{a}}}\;,$$

where *E* is the elastic modulus, *h* is the vessel wall thickness, *ρ* is the blood density, *r* is the vessel radius, *A* is the vessel area, and *C*_*a*_ is the vessel compliance. The Bramwell‐Hill equation will be used in this parameter estimation approach.

It should be noted that the targeted pulse pressure and CO are taken directly from patient measurements and the pulse pressure in the model primarily depends on the arterial compliance and the CO. As a result of the compliance term *C*_*a*_ being determined from the measured pulse pressure, the *C*_*a*_ in the Bramwell‐Hill equation is considered a known value and thus only the area is adapted to converge to the PWV. As the blood density *ρ* is considered constant and consistent across all simulations, the PWV will be primarily related to the vessel areas between the brachial artery and the posterior tibial artery.

The arterial compliances ${Ĉ}_{a}$ and vessel areas Â from the modelling results in tier 1, in combination with the PWV obtained from the measurements, are used to predict the new vessel areas of the main systemic vessels in tier 2. Given that the ratio of PWV can be written as

(2) $$\frac{PWV^{n+1}}{PWV^{n}}=\frac{\left(\sqrt{\frac{{Â}^{n+1}}{\rho {Ĉ}_{a}}}\right)}{\left(\sqrt{\frac{{Â}^{n}}{\rho {Ĉ}_{a}}}\right)}\;\approx \frac{{\sqrt{{Â}^{n+1}}}_{1}}{{\sqrt{{Â}^{n}}}_{2}}=\sqrt{\frac{{Â}^{n+1}}{{Â}^{n}}}=\sqrt{\Psi },\;\Rightarrow \;{Â}^{n+1}=\Psi {Â}^{n},$$

where the subscript ^*n*^ denotes the value from the previous cardiac cycle and ^*n*+1^ denotes the areas that are required in the model to achieve the same PWV as the measurement. It should be noted that the compliances from the brachial artery to posterior tibial artery are assumed to be approximately the same between the previous and current cardiac cycle. As a result, the area ratio Ψ then gives an estimation on how much the mean vessel areas from the previous cardiac cycle need to increase (or decrease) in order to converge the PWV of the model simulation toward the measured PWV. In the model, the reference areas *A*_*d*_ of the main systemic vessels are modified in order to converge to the measured PWV.

The final stage of the parameter estimation is performed by adapting the uterine vessel areas of the model to converge to the measured uterine artery velocity waveform obtained via pulse Doppler ultrasound. The areas of the uterine arteries and arcuate arteries will be denoted by ${Â}_{ut}$, while the areas of the spiral arteries is denoted by ${Â}_{spiral}$. Iterative adaptation of the vessel areas, ${Â}_{ut}$ and ${Â}_{spiral}$, and the vascular bed resistances of the uterus and placenta, ${\hat{R}}_{beds}$, is then achieved based on the end‐diastolic, $U_{ED}^{model}$, and peak‐systolic, $U_{sys}^{model}$, model uterine artery velocities from the previous cardiac cycle according to the following algorithm:

- IF ( $U_{ED}^{model}$ > $U_{ED}^{measured}$ & $U_{sys}^{model}$ > $U_{sys}^{measured}$) THEN (reduce ${Â}_{ut}$, reduce ${Â}_{spiral}$, increase ${\hat{R}}_{beds}$)
- IF ( $U_{ED}^{model}$ < $U_{ED}^{measured}$ & $U_{sys}^{model}$ < $U_{sys}^{measured}$) THEN (increase ${Â}_{ut}$, increase ${Â}_{spiral}$, reduce ${\hat{R}}_{beds}$)
- IF ( $U_{ED}^{model}$ > $U_{ED}^{measured}$ & $U_{sys}^{model}$ < $U_{sys}^{measured}$) THEN (increase ${Â}_{ut}$, reduce ${Â}_{spiral}$, increase ${\hat{R}}_{beds}$)
- IF ( $U_{ED}^{model}$ < $U_{ED}^{measured}$ & $U_{sys}^{model}$ > $U_{sys}^{measured}$) THEN (reduce ${Â}_{ut}$, increase ${Â}_{spiral}$, reduce ${\hat{R}}_{beds}$)

Hence, it was assumed that the spiral arteries and resistive beds predominantly determine the end‐diastolic velocities, while the peak systolic uterine velocities are predominantly affected by the areas of the uterine and arcuate vessels.

### 2.5 Wave separation and wave intensity analysis

There are two main approaches typically used to mathematically analyse wave propagation in 1D cardiovascular modelling. The more traditional method of analysing signals and waves is through Fourier analysis, which is performed in the frequency domain and relies on representing a waveform as a series of sinusoidal functions whose frequencies form a harmonic series; an alternative technique is wave intensity analysis (WIA),^59^, ^60^ which allows a wave to be split into forward and backward propagating components and is performed in the temporal domain. One of the main shortcomings of Fourier analysis is that it is generally not possible to relate a specific harmonic to a particular point event time as one or multiple cardiac cycles are utilised to calculate the spectrum of frequencies. On the other hand, as WIA is performed in the time domain, WIA can be easily utilised to study a specific event in time. Thus in this work, WIA is utilised to investigate wave transmission and reflection phenomena in the cardiovascular network and determine what causes specific uterine velocity waveform characteristics.

As wave intensity is mathematically intensive, only the final equation representation is described. A full description of the methods derivation can be found in previous studies.^49^, ^60^ The velocity (U) and pressure (P) waveforms are composed of forward (+) and backward (‐) components,^49^, ^61^ which allows the combined wave to be separated represented as

(3) $$dP=dP_{-}+dP_{+},\;\;dU=dU_{-}+dU_{+},$$

where the forward and backward components can be calculated as

(4) $$dP_{\pm }=\frac{1}{2}\left(dP\pm \rho cdU\right),\;dU_{\pm }=\frac{1}{2}\left(dU\pm \frac{dU}{\rho c}\right),$$

where *c* is the wave speed. The time‐corrected wave intensity (*wi*) can be be described by

(5) $$wi_{\pm }=\frac{\pm }{4\rho c}{\left(\frac{dP}{dt}\pm \rho c\frac{dU}{dt}\right)}^{2}.$$

One of the strengths of WIA is its inherent ability to determine whether a wave is travelling in the forward or backward direction and whether it is compressive or expansive. This can help indicate whether a characteristic in a velocity or pressure waveform is caused by a forward or backward propagating wave, although it is generally not possible to find the exact origin of the wave reflection.

## 3 RESULTS AND DISCUSSION

### 3.1 Model uterine artery waveforms

**FIGURE 3:**
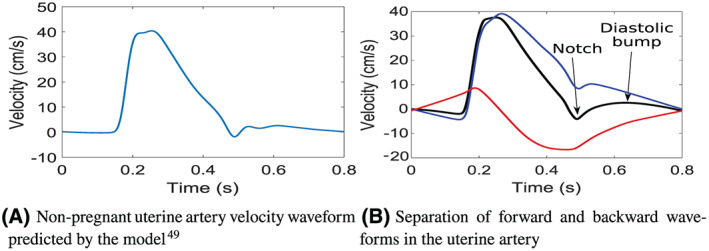
Left uterine artery velocity waveforms for non‐pregnant example in A, and the separation of forward and backward velocity waves in B,

A typical nonpregnant velocity waveform can be seen in Figure 3A, which is generated from the model described by Carson and Van Loon.^49^, ^50^ During systole, the waveform is quite sharp, while a notch is generally present in the waveform at the beginning of diastole, and there is close to zero flow during the entirety of diastole, indicating a high resistance and low compliance of the uterine vascular bed during nonpregnant conditions. The notch typically remains in early pregnancy, but in the majority of cases, it tends to be dampened out during the second trimester, primarily due to an increased arterial compliance. In a healthy pregnancy, the adaptation of the uterine vessel diameters and compliances, total vascular resistance, total arterial compliance, and blood volume occurs very quickly in early pregnancy while slows down significantly as pregnancy progresses toward the mid‐second trimester and third trimester.

**FIGURE 4:**
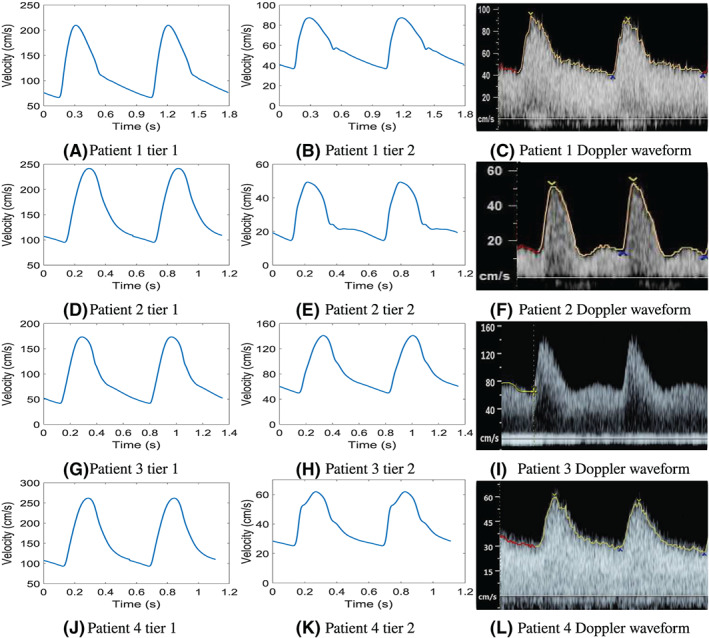
Left uterine artery waveforms for all patients. Results from parameter estimation tier 1 for patients 1 to 4 are on the left side in A, D, G, J, parameter estimation tier 2 in the centre in B, E, H, K, and the measured Doppler waveforms on the right in in C, F, I, L

Figure 4 compares the left uterine artery velocity waveform predicted by the model for tiers 1 and 2 of the parameter estimation strategy and also the measured Doppler waveforms of each patient. As discussed in section 2.3, it is important to realise that the waveform shape is more important than the magnitude of velocity, partly due to the limitations of Doppler ultrasound (as discussed in section 2.3), and as the Doppler waveforms utilised are for the maximum velocity detected in the cross‐section, while for the model the velocity is chosen to be *v*=*Q*/*A*, which is the mean velocity in the cross‐section T.

The waveform shape predicted for patient 1 is very similar for the first and second tiers of the parameter estimation; however, there is a substantial difference in magnitude of the velocity between the waveforms. The waveform predicted in tier 1 does not have the early‐diastolic notch that is present in tier 2. The model‐predicted waveform in Figure 4B shows a strongly defined pulse that has a very similar shape to that extracted by Doppler and is shown in Figure 4C. The waveforms have a relatively smooth, defined pulse during systole with significant blood velocity observed during diastole. The maximum diastolic velocity occurs at the beginning of diastole, while there is a slight decreasing trend in the velocity to the end of diastole. An early diastolic notch is observed in the model‐predicted waveform, but due to noise (which is common in ultrasound), it is not entirely clear if this notch is present in the Doppler measurements. The pulsatility index predicted by tier 2 of the model is slightly overestimated in comparison with the Doppler waveform.

For patient 2, there are significant differences in the uterine artery velocity waveform between tiers 1 and 2 (Figure 4D,C). The first tier significantly overestimates the magnitude of velocity in the uterine arteries, while the waveform has a much smoother shape. The uterine velocity waveform for tier 1 has a much lower rate of descent in diastole compared with that of tier 2 or the Doppler waveform (Figure 4F). The tier 2 uterine artery velocity waveform is much closer to the shape and, unsurprisingly, magnitude of the Doppler velocity waveform. The tier 2 model‐predicted waveform includes a notch in the waveform in early diastole and a much sharper, more defined pulse that is indicative of high uterine resistance and low compliance. The notch in the Doppler waveform is much more pronounced compared with that of the model prediction; nevertheless, the model does give a good representation of the overall shape of the uterine artery velocity waveform. The pulsatility index is underestimated by the model when compared with the Doppler waveform.

The velocity waveforms of patient 3 for tiers 1 and 2 (Figure 4G,H) have similar shapes but the diastolic velocity is underestimated while the peak systolic velocity is overestimated for tier 1. Both of the model‐predicted waveforms are very smooth over systole without a notch present in the waveform in early diastole. Tier 2 shows a stronger resemblance to the Doppler waveform (Figure 4I) than tier 1. At the end of diastole, the tier 2 velocity waveform has a slight downward notch just before systole which can also be observed in the Doppler waveform. The Doppler waveform pulse is much sharper compared with the model with a notch present in early diastole, and the velocity then increases slightly to mid‐diastole, before reducing again until the beginning of systole. Although the model does not capture the notch that is observed in diastole in the Doppler waveform, it predicts a pulsatility index very close to that of the Doppler measurement.

For patient 4 (Figure 4J‐L), the model‐prediction after tier 1 still vastly overestimates both end‐diastolic and peak systolic velocities. The optimisation in tier 2 accounts for this to bring the results closer to the Doppler measurements, but the adaptations of the utero‐ovarian system in this second tier also cause some distinct changes to the shape of the waveform. A deceleration of rate of change can be seen during the systolic phase. This characteristic might be identified in the Doppler data, but it is certainly not as pronounced and obscured by the tracing. Similarly, a subtle kink can be seen in diastole around *t*=0.4, which can not be clearly identified in the Doppler data. However, the differences observed between the tiers 1 and 2 results for this patient stress the importance of the uterine system on the shape of the developed waveforms.

### 3.2 Area of the main uterine vessels

The vessel diameters predicted by the model are within the expected range (large variations) given in literature^28^, ^64^, ^65^, ^66^, ^67^, ^68^, ^69^, ^70^ (shown in Table 3). The model predicts that the spiral arteries of patient 2 are significantly under‐adapted compared with the other patients and to what is expected from literature. This indicates that patient 2 would have significantly higher resistances in the uterine region compared with the other patients. In addition, the predicted diameter of the spiral arteries was 0.27 *mm* for patient 2, who developed pre‐eclampsia later in pregnancy, this is close to observations in Brosens et al,^71^ where the mean diameter of the spiral arteries was 0.2 *mm* for pre‐eclamptic patients and 0.5 *mm* in healthy pregnancies. However, this is contrasted by patient 1 who also developed pre‐eclampsia later in pregnancy where the diameter was predicted to be 0.84 *mm*, although patient 1 had a normal Doppler waveform. The predicted spiral artery diameter for patients 3 and 4 were 0.46 and 0.52 *mm* respectively, which are expected for a healthy pregnancy at mid‐gestation.

### 3.3 Pulse wave velocity

The brachial‐ankle pulse wave velocities measured from the patients are shown in Table 2, while the model PWV values are shown in Table 3. The PWV estimates of the model for parameter estimation tier 2 converged well to that of the measured data. Rather than analysing the PWV directly, this section will investigate the area ratio change $\hat{\Psi }$ of the main systemic vessels (vessels from the aorta to the brachial artery, and also the aorta to the posterior tibial artery) given in equation 2from parameter estimation tiers 1 and 2. The area ratio changes between tiers 1 and 2 in the model are shown in Table 3.

**TABLE 2:** Patient measurement data

Patient	Height	Weight	GAM	SBP	DBP	HR	CO	PWV	
	(cm)	(kg)	(Week + Day)	SBP (mmHg)	DBP (mmHg)	HR (bpm)	(L/min)	(m/s)	PI
1	164	85	25+1	103	65	109	6.1	7.7	0.77
2	166	80	25+2	131	93	104	7.3	9.4	1.77
3	156	80	22+0	124	78	89	3.5	7.3	0.8
4	165	80	23+6	136	92	108	7.3	6.6	0.69

*Note*. Height is in centimetres, week is the gestational week at the time of the measurements, SBP and DBP are the systolic and diastolic blood pressures in mmHg, HR is the heart rate in beats‐per‐minute, CO is the cardiac output in *L*/*min*, PWV is the pulse wave velocity in *m*/*s*, and PI is the pulsatility index in the uterine artery. Abbreviation: GAM, gestational age at measurement.

**TABLE 3:** Overview of model results

Patient	PWV 1	PI 1	PWV 2	PI 2	$\hat{\Psi }$	UA (mm)	Arc (mm)	R/S (mm)
1	6.904	1.20	7.701	0.869	1.31	1.55	4.95	0.84
2	7.870	0.970	9.318	1.241	1.69	1.44	3.51	0.27
3	12.093	1.424	7.256	0.682	0.42	1.79	2.26	0.46
4	8.572	1.06	6.605	0.916	0.56	1.25	1.67	0.52
Vessel Area range from Literature	‐	‐	‐	‐	‐	1.18^62^ to 3.0^28^	0.5^63^ to 6.0 (double uterine vessel size at term)^64^, ^65^	0.2^66^ to 3.0^67^

*Note*.The pulse wave velocity is measured in *m*/*s*, PI is the pulsatility index, and $\hat{\Psi }$ is the area ratio for the main systemic vessels, ie, $\hat{\Psi }=\frac{A_{tier2}}{A_{tier1}}$. The final model‐predicted diameters at the midpoint of the uterine artery (UA), arcuate artery (Arc), and radial/spiral arteries (R/S). For comparison purposes, the range of vessel areas from literature are also presented.

For patients 1 and 2, both of whom developed pre‐eclampsia in later pregnancy, the vessel areas of the main vessels (tier 1) needed to be increased for the second parameter estimation strategy in order to achieve pulse wave velocities close to the measured values. This is in agreement with a study in which the PWV in mice has been observed experimentally to have a positive linear relationship with vessel diameter^72^ and agrees with the expected relationship via the Bramwell‐Hill equation 1. Another study investigated the ascending aorta and aortic arch diameter in relation to hypertension and found that, in general, hypertensive patients had much larger diameters^73^ and the increased blood pressure was unlikely to account for this increased diameter alone. Furthermore, it was observed in Easterling et al^74^ that over the course of a normal pregnancy, the aortic diameter increased significantly in size and it was larger for pre‐eclamptic women than in normotensive. However, the diameters were only known for those women with pre‐eclampsia and not for the women that were normotensive but develop late‐onset pre‐eclampsia. Thus, it is not possible to clearly state that aortic diameter in itself is an indicator of risk to develop a hypertensive disorder, or if the increased vessel diameter in these patients is linked to poor adaptation or poor chemical/hormonal regulation.

In comparison, patients 3 and 4 both had normal pregnancy outcomes. For these two patients, the areas of the main systemic vessels from parameter estimation tiers 1 and 2 decreased. The estimated PWV of patients 1 and 3 are quite similar, but due to the model's compliance estimate which converges to the pulse pressure, the mean vessel area at diastole in the main systemic arteries of patient 1 is estimated to be 2.97 times larger than those of patient 3.

The findings of this section indicate that in order to estimate arterial stiffness, one must consider a combination of PWV and pulse pressure as each measure alone is not enough to suggest elevated levels of arterial stiffness.

### 3.4 Separation of waveforms into forward and backward propagating components

**FIGURE 5:**
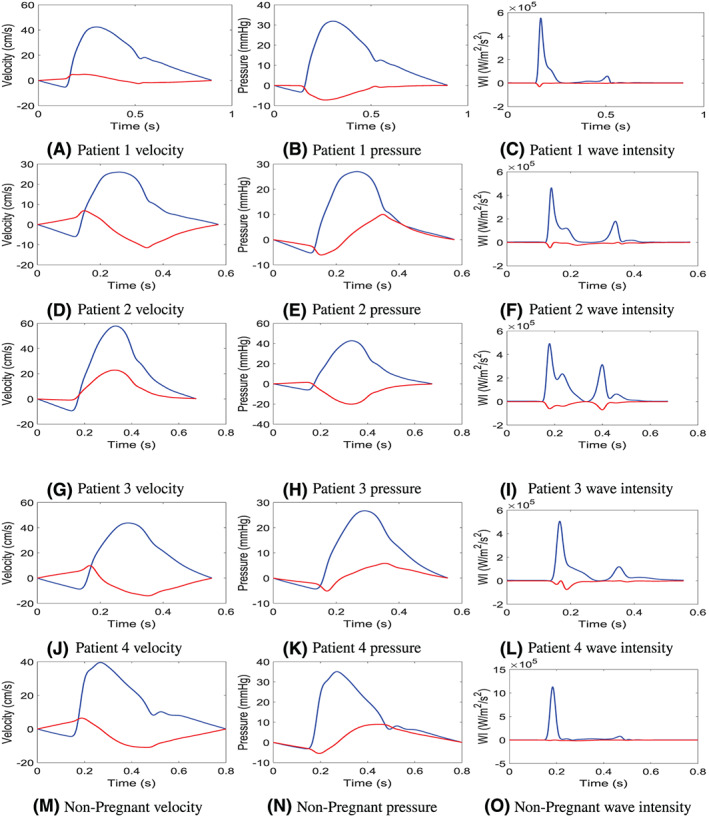
Wave separation of velocity, pressure, and wave intensity in the left uterine artery where the blue line represents the forward propagating wave and the red line is the backward propagating wave. Shown for patient 1 in A, B, and C,; for patient 2 in D, E, and F,; for patient 3 in G, H, and I,; for patient 4 in J, K, and L,; and for a nonpregnant case form, the model of Carson and Van Loon^49^, ^50^ in M, N, and O

At any point in the cardiovascular network, a velocity and pressure waveform is a combination of all forward‐ and all backward‐propagating waves. Therefore, it is important to consider the individual effects these forward‐ and backward‐propagating components have on the overall waveform. As clinical measurements are only capable of measuring the combined waveform, a model could significantly aid the analysis of these waves by investigating how and where these reflected waves occur. Figure 5 shows the forward‐ and backward‐propagating velocity, pressure, and wave intensity in the left uterine artery for all patient cases, as predicted by the model using the parameter estimation tier 2. The backward‐travelling waves are caused by reflections from the uterine circulation, while the forward‐travelling waves are from all upstream vasculature that includes the rest of the systemic arterial system. It is useful to note that the shape of the forward travelling velocity wave is similar to the shape of the forward travelling pressure, while the shape of the backward travelling velocity is similar in shape to the backward travelling pressure inverted over the x‐axis. The results clearly indicate that a notch is formed from a combination of reflections from the uterine circulation and the rest of the systemic circulation. In particular, a notch forms if

- during diastole, the gradient of the backward travelling velocity is positive, ie, $\frac{\partial U_{-}}{\partial t}>0$;
- the gradient of the forward travelling velocity is negative, ie, $\frac{\partial U_{+}}{\partial t}<0$; and
- the absolute gradient of the backward travelling velocity is greater in magnitude than the absolute gradient of the forward travelling wave, ie, $\left|\frac{\partial U_{-}}{\partial t}\right|>\left|\frac{\partial U_{+}}{\partial t}\right|$.

The backward‐travelling velocity and pressure waves for patients 1 and 3 are similar in shape, and the wave reflection causes a negative pressure that propagates backwards, which creates a suction‐like effect in the uterine arteries and hence the backward wave actually increases the blood velocity.

The backward‐travelling waves for patients 2, 4, and the nonpregnant example waveform show similar characteristics. The wave reflection initially causes a negative pressure that propagates backwards and creates a suction‐like effect to increase the blood velocity; however, the wave reflection eventually creates a backward‐travelling wave front of positive pressure, which means the blood needs to travel against a larger pressure gradient. At this point, the contribution of the backward running wave is to create a negative velocity which is most likely created by a larger downstream resistance in the uterine system.

### 3.5 Uniqueness of model solutions

This section serves to test the main assumptions made in the parameter estimation algorithm. During the first estimation technique, the diameters of the uterine arterial circulation are based on reference values from literature.^50^ The first parameter estimation tier estimates the volumetric inflow rate at the aortic root which is then used as the inlet boundary condition in the second parameter estimation tier. In the second tier, only the systemic arteries are modelled while the diameters of the three main vessels in the uterine circulation (uterine artery, arcuate artery, and spiral artery) are adapted to achieve velocity waveforms similar to that measured by Doppler ultrasound. This leaves two main questions to investigate:

- Are the solutions of the proposed two‐tiered parameter estimation self‐consistent between the tiers?
- Do different diameter variations of the uterine vessels create unique solutions?

To investigate question 1, we performed an additional simulation for each patient that essentially reruns parameter estimation tier 1 but now uses the final uterine artery vessel diameters that were predicted from tier 2 instead of the reference diameters. This allows us to investigate whether a change in the uterine artery vessel sizes will change the predicted aortic root flow rate waveform. As flow rates in diastole are close to zero the following relative errors are utilised:

(6) $$\text{maximum error}=\epsilon_{max}=\frac{\mathrm{Max}\left|\left(Q_{a}^{i}-Q_{b}^{i}\right)\right|}{\mathrm{Max}(Q_{a}^{i})},$$

(7) $$\text{mean error}=\epsilon_{mean}=\frac{\text{Mean}\left|\left(Q_{a}^{i}-Q_{b}^{i}\right)\right|}{\mathrm{Max}(Q_{a}^{i})},$$

where *Q*_*a*_ is the aortic inflow from tier 1 when using the reference uterine vessel diameters, *Q*_*b*_ is the aortic inflow when using the uterine vessel diameters predicted from tier 2, and superscript *i* (*i*=1,2,…,*t*) represents the value of the inflow rate vector Q for the *i*th time step, and *t* is the maximum number of time steps. The errors for each patient case are shown in Table 4. All results show less than a 1*%* difference, which is smaller than the convergence tolerance used during the simulations. This shows that using the aortic inflow predicted via parameter estimation tier 1 with the reference uterine vessel diameters is a valid model condition.

**TABLE 4:** Comparison of the converged aortic inflow prediction for parameter estimation tier 1 when using the reference uterine vessel diameters and the uterine artery vessel diameters predicted from the parameter estimation tier 2

	Patient 1	Patient 2	Patient 3	Patient 4
*ϵ*_*max*_(‐)	3.1×10^−4^	3.9×10^−3^	8.1×10^−4^	5.1×10^−3^
*ϵ*_*mean*_ (‐)	8.7×10^−4^	9.9×10^−4^	7.9×10^−4^	8.5×10^−4^

**FIGURE 6:**
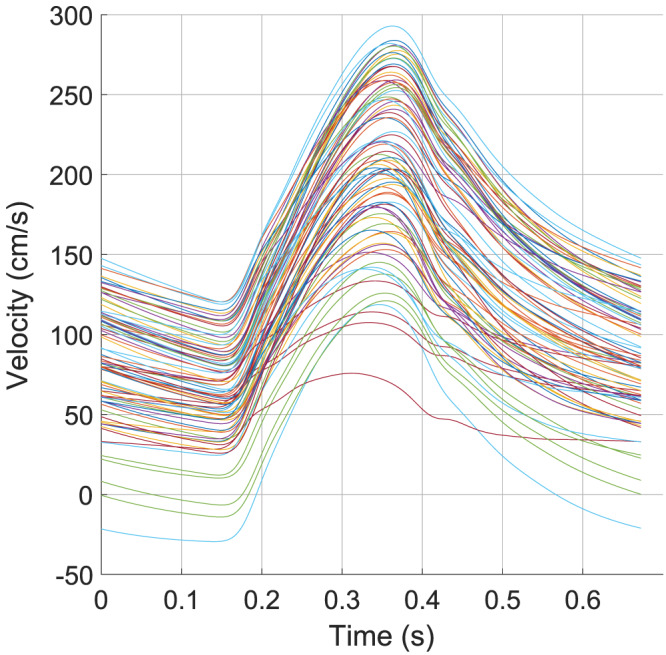
Plot of all solutions obtained via the Monte‐Carlo simulation. Each solution was generated using a different combination of uterine artery, arcuate artery, and spiral/radial artery diameters

In the second part of this uniqueness test, a Monte‐Carlo simulation for patient 3 is presented (patient 3 was chosen at random). To this end, the open‐loop arterial network from parameter estimation tier 2 was used with the aortic inflow boundary condition predicted via tier 1. However, no optimisation of the uterine vessel diameters was performed, but instead, a randomised independent set of variations was determined for the uterine artery vessel diameters in order to investigate whether each variation would produce a unique velocity waveform. The range of uterine vessel areas for these simulations was $\left[\frac{A_{ref}}{4},4A_{ref}\right]$ with a uniform random distribution in this range, where *A*_*ref*_ is the reference area of the vessel (Table 5). A total of 225 simulations were performed under these conditions to give a good spread of uterine vessel areas. Figure 6 shows all the solutions obtained via the Monte‐Carlo simulation. Analysis of these waveforms and uterine vessel areas indicate that the spiral arteries in the model primarily affected the downstream resistance, which has the impact of moving the entire velocity waveform up or down, but did not significantly change the waveform shape. The uterine artery diameter was mainly responsible for changing the pulse velocity, defined as the difference in systolic and diastolic velocity values. It was also observed that there was no value for uterine vessel diameter that caused a full notch to form in the velocity wave, instead the arcuate arteries and uterine artery primarily enhanced, or reduced the characteristics of the waveform that was produced from the main systemic arteries. This indicates that it is likely not the uterine vessels alone that cause the notch in the velocity waveform and that the entire arterial system must be considered. Finally, waveforms that were similar in shape and magnitude had similar diameters for uterine vessels, implying that each combination of these uterine vessel areas gives rise to a unique solution, with the assumption that all other modelling aspects, such as CO, remain constant across the Monte‐Carlo simulations. This was tested more quantitatively through the following process:

- The model‐predicted peak systolic and end‐diastolic velocities in the left uterine artery were extracted from the solutions.
- For each simulation case, a minimisation procedure was used to find the closest neighbouring solution for the peak systolic and end‐diastolic velocities.
- The full velocity waveform and the uterine artery sizes were then compared between the closest neighbour solutions.

**TABLE 5:** The area reference values for the uterine, arcutate, and radial/spiral arteries

	Uterine	Arcuate	Radial/Spiral
*A*_*ref*_ (cm^2^)	0.064	0.1169	0.0041

### 3.6 Model limitations

In this work, no information on vessel geometry was known, which includes vessel length, areas, and connectivities. Thus, due to lack of data, a general network that uses the most common anatomical configuration of uterine vessels^49^, ^50^ is used in this work. This could have an impact on wave‐reflections due to impedance changes at different locations in the network. In order to fully validate the parameter estimation technique involving PWV, a future study should ideally include several pieces of vessel information, in particular the brachial artery, aorta, femoral artery down to the posterior tibial artery, external and internal iliac artery, and the left and right uterine arteries.

The uterine artery velocity waveform measured by Doppler ultrasound can show important characteristics in shape, such as early diastolic notching and indices like the pulsatility index and resistance index; however, the magnitude of these velocities can be misleading as Doppler only measures the maximum velocity in the cross‐section. This highly depends on the velocity profile which can vary significantly between individuals and even varies at the same location at different points in the cardiac cycle. Concerns regarding the use of Doppler ultrasound have been discussed by Blanco,^53^ particularly in regards to the common carotid artery.

The small cohort utilised in this study must be expanded in the future in order to further test the findings of this work.

## 4 CONCLUSIONS

A framework was developed to integrate noninvasive clinical data on pregnant women into a “template” cardiovascular network model to create patient‐specific models. The approach sought to estimate global systemic parameters alongside local uterine quantities in an attempt to establish a more complete physiological picture that can help explain uterine waveforms from a mechanistic perspective. The model predicted that the pregnant patients who were known to develop pre‐eclampsia later in pregnancy had larger vessel diameters (see $\hat{\Psi }$ in Table 3) than those who had normal pregnancy outcomes. Furthermore, a comparison of the two tiers proposed suggests that estimations of arterial stiffness should include both PWV and pulse pressure measurements, as individually, they do not inherently indicate increased arterial stiffness, which is a combination of both vessel area and vessel compliance/distension. The results indicate that the presence of a notch in the uterine artery is strongly dependent on both the downstream vasculature (uterine circulation) and the upstream vasculature (the rest of the systemic system). Hence, in order to predict the presence of this notch, conditions in the entire systemic circulation must be considered.
